# Cleaning Efficacy, Debris Extrusion, and Instrument Separation during Root Canal Filling Removal using Continuous Rotation, Reciprocation, and Optimum Torque Reverse Motions: An *In Vitro* Study

**DOI:** 10.1055/s-0045-1814775

**Published:** 2026-01-20

**Authors:** Tarek Abboud, Mouhammad Al-Tayyan, Hassan Achour, Yasser Alsayed Tolibah

**Affiliations:** 1 Department of Operative and Endodontic Dentistry, Faculty of Dentistry, Damascus University, Damascus, Syria; 2 Department of Endodontics, Faculty of Dentistry, Damascus University, Damascus, Syria; 3 Department of Pediatric Dentistry, Faculty of Dentistry, Damascus University, Damascus, Syria

**Keywords:** endodontic retreatment, OTR motion, continuous rotational motion, reciprocating motion

## Abstract

**Objectives:**

This study aims to address the Optimum Torque Reverse (OTR) efficacy in root canal obturationmaterials removal, the amount of apically extruded debris, and separating instruments (SI) as a complication compared to continuous rotational motion (CRM) and reciprocating motion (RM).

**Materials and Methods:**

The sample involved 45 straight mandibular first premolars. The crowns were decoronated to standardize the length to 16 mm. The root canals were prepared up to a size 25 taper 0.06, obturated using the lateral compaction technique with a resin-based sealer, stored for 7 days, and divided according to the motion type used during retreatment into three groups (each group consisted of 15 samples); Group 1: CRM using Protaper Universal Retreatment system (D3 20 0.07 last file reach apical third), Group 2: RM using WaveOne Gold system Medium 25 0.07 variant taper, and Group 3: OTR motion using Protaper Universal Retreatment system (D3 20 0.07 last file reach apical third). The extruded debris weight was measured using an analytical balance. Samples were sectioned longitudinally and photographed at 20X magnification using an optical microscope. The images were analyzed in ImageJ to assess the percentage of residual obturation material. The number of SI during retreatment was recorded. Statistical Analysis Data were statistically analyzed using one-way analysis of variance (ANOVA) and chi-square tests at an α = 0.05 significance level.

**Results:**

The results indicate that the incidence of SI during retreatment in the OTR motion group was higher than the CRM group and the RM group ( *p* = 0.001). There were no significant differences among the groups regarding the amount of apically extruded debris and the percentage of residual obturation material ( *p* >0.05).

**Conclusion:**

The effectiveness of root canal obturation material removal and the amount of extruded debris during retreatment do not depend on the motion used. However, the OTR motion is associated with an increased risk of SI during retreatment.

## Introduction

Endodontic treatment often fails due to persistent or recurrent infection resulting from insufficient cleaning, shaping, or obturation of the root canal system. As a result, nonsurgical endodontic retreatment is considered the preferred therapeutic approach. 1

The ProTaper Universal Retreatment system comprises three files: D1, D2, and D3, each featuring a convex triangular cross-section specifically engineered to aid in the removal of obturation materials. The flute design of these files is optimized to clear residual debris during clockwise (CW) rotation 2 effectively.

In 2008, Yared proposed a new concept of reciprocating motion (RM). 3 Following this, manufacturers introduced single-file reciprocating systems such as WaveOne Gold (Dentsply Maillefer, Ballaigues, Switzerland), which are heat-treated during manufacturing to enhance rotational fatigue resistance and increase instrument flexibility. It consists of four file sizes: Small (20/0.07), primary (25/0.07), medium (35/0.06), and large (45/0.05). All these files have a parallelogram cross-section with an off-center rotational axis and an 85-degree cutting-edge angle in contact with the canal walls, featuring a decreasing variable taper. 4

A new preparation technique was developed, involving the Elements Motor (SybronEndo, Orange, CA) and the TF Adaptive® file system (TFA) by SybronEndo (Orange, CA). This unique, patented motion automatically adapts to the stress applied to the instrument within the canal. 5 It aims to combine the advantages of both continuous rotation motion (CRM) and RM, reducing the risk of instrument separation within the canal without compromising performance. 5

In-line with this trend, J Morita (Kyoto, Japan) developed the Optimum Torque Reverse (OTR) motion to exploit the benefits of RM while minimizing its drawbacks. 6 OTR motion has shown greater resistance to cyclic fatigue than continuous rotary motion, while maintaining optimal cutting efficiency. 7 8 Notably, according to the manufacturer, the OTR motion can be used with all files designed for continuous rotation. 9

When using OTR motion, the file starts with continuous rotation, and torque is measured continuously. If the torque is below a preset value, the file continues to rotate CW. If the torque reaches the preset value, the file rotates counterclockwise by 90 degrees, then rotates CW by 180 degrees until the torque drops below the preset value. 8 Thus, OTR motion involves partial reciprocation with an overall CW rotation, making it compatible with instruments designed for cutting efficiency in CW rotation (continuous rotational motion [CRM] instruments). 7

Previous studies have investigated the efficacy of adaptive motion—which combines continuous rotary and reciprocating movements—during the removal of root canal obturation materials, as well as its influence on debris extrusion and cyclic fatigue resistance during retreatment. While one study reported that adaptive motion was more effective than rotary motion in removing obturation materials, another found that continuous rotary motion was superior. In terms of debris extrusion, RM was shown to produce significantly more debris compared to rotary motion, whereas no significant differences were observed between rotary and adaptive motions. Furthermore, regarding cyclic fatigue resistance, ProTaper Next instruments experienced separation during RM, but none occurred during continuous rotary motion. 2 10 11

In a previous study, 12 a comparison between Endostar E3 Azure and EdgeFile X7, with both continuous rotary and OTR motion, was conducted; it was reported that OTR motion reduced apical debris extrusion during canal treatment. In another investigation, 13 the amount of apically extruded debris was compared among various motion kinematics, including OTR, TFA, continuous rotation, reciprocation motion, and Jeni motion, during the preparation of moderately to severely curved root canals. The findings revealed that the Jeni motion exhibited a similar amount of apically extruded debris as the OTR motion and significantly less than the other tested motion types.

Despite the ongoing development of rotary kinematics—including continuous rotation, reciprocation, adaptive motion, and, more recently, OTR—substantial gaps remain in the literature regarding their comparative effectiveness during nonsurgical retreatment. Recent investigations have demonstrated that OTR motion can reduce torque and apically directed force during canal preparation while improving the cyclic fatigue resistance of Nickle Titanium (NiTi) instruments, suggesting a potential biomechanical advantage over conventional continuous rotation and reciprocation systems. 9 14

Therefore, a comprehensive evaluation of OTR motion—compared with CRM and RM—is warranted to determine whether OTR offers a balanced advantage by improving obturation-material removal efficiency, minimizing apically extruded debris, and enhancing torsional and fatigue resistance during retreatment. To address these gaps, the present study aims to provide an evidence-based assessment of OTR motion across clinically relevant parameters. The null hypothesis proposed that all tested motion kinematics would demonstrate similar effectiveness in obturation material removal, apical debris extrusion, and torsional fatigue resistance.

## Materials and Methods

### Ethical Statement

This *in vitro* experimental study received ethical approval from the Local Research Ethics Committee of the Faculty of Dentistry, Damascus University (UDDS-76-20122021/SRC-1428), and was funded by Damascus University. This study was conducted in accordance with the Helsinki Declaration.

### Sample Size Calculation

Based on changes in the percentage of residual obturation material across five rotary file systems, 15 the sample size was calculated using G Power 3.1.9.4. For the analysis of variance(ANOVA) test, 15 samples per group (45 in total) were required to detect an effect size of 0.52 with 85% power at a 0.05 significance level.

### Sample Selection and Preparation

Freshly extracted, intact, mature mandibular first premolars, removed for orthodontic or periodontal reasons, were included in the study. These premolars were collected from the Departments of Orthodontics and Oral and Maxillofacial Surgery at the Faculty of Dentistry, Damascus University. After extraction, the premolars were examined with a 2.5-magnification lens (Carson handheld, Ronkonkoma, NY) to ensure they were free from cracks. Any visible calculus was removed using ultrasonic tips (Eighteeth, Changzhou, China). Two periapical radiographs (buccolingual and mesiodistal) using intraoral sensor (Ez Sensor HD, VaTech, South Korea) were taken to each premolar to ensure that it was at Vertucci type I and free from anatomical abnormalities ( Fig. 1 ). Finally, the teeth were kept for 1 minute in 5.25% NaOCl (Shahabamed, Aleppo, Syria), and then stored in normal saline. 16 Premolars with root resorption, root curvature (more than 10 degrees), apical minor constriction greater than a size #25 K-file, calcified canal, fracture, or previous obturation were excluded.

**Fig. 1:**
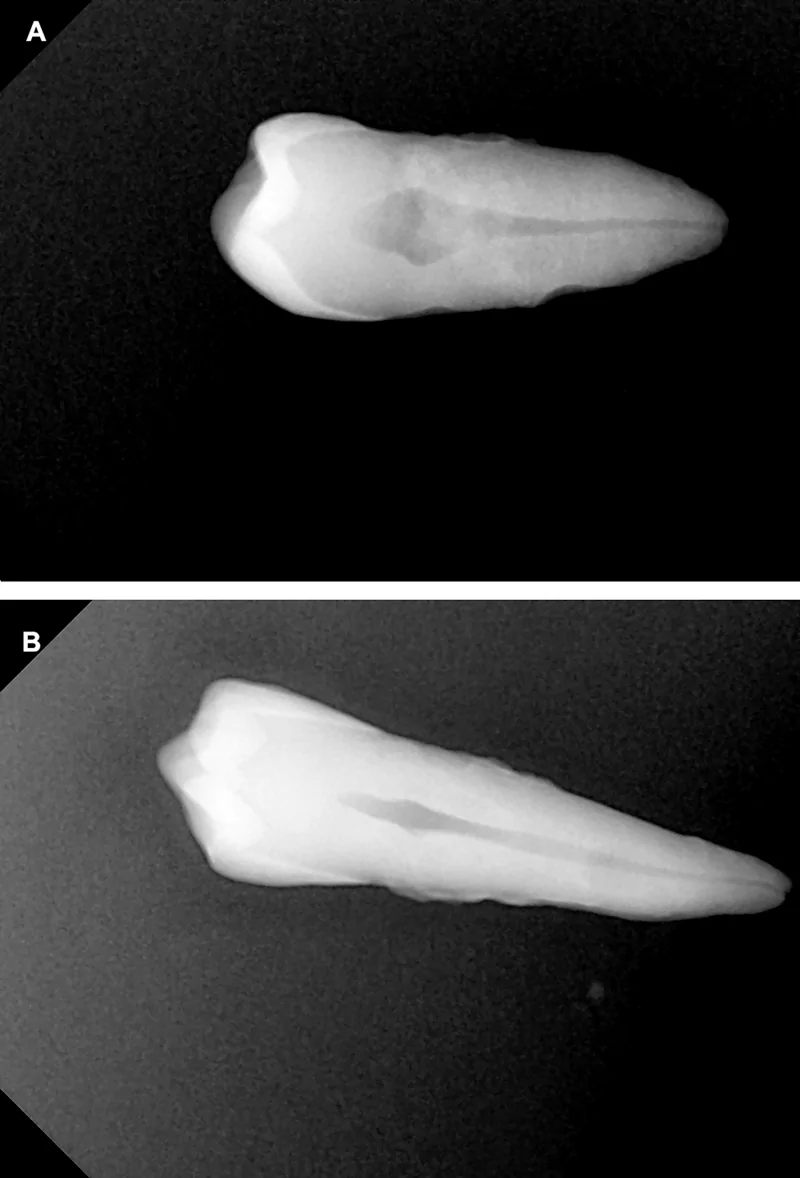
Diagnostic periapical radiographs. ( **A** ) Buccolingual direction, and ( **B** ) mesiodistal direction.

Forty-five premolars were decoronated at 16 mm from the apex by a double-faced diamond disk (Hager & Meisinger, Neuss, Germany), mounted on a low-speed straight handpiece to standardize sample lengths and facilitate canal instrumentation. 17 The patency was checked with a 10 K-file (Mani, Utsunomiya, Japan) until the tip was visible at the respective apex, and the working length (WL) was established by subtracting 1 mm from this measurement.

### Root Canal Treatment and Obturation

Premolars were prepared by a single practitioner (T.A.) using Fanta rotary Ni-Ti instruments up to a size of 25 with a 6% taper (25/0.06) for root canal treatment. The preparation followed the sequence in the AF Gold system (Fanta Dental, Shanghai, China). Approximately 2.5 mL of 5.25% NaOCl was used for irrigation between files, followed by a final irrigation with 5 mL of 17% EDTA MD-Cleaner (Meta Biomed, Chungcheong Buk-do, South Korea) for 30 seconds, and then rinsed with 5 mL of normal saline. The irrigants were applied using a 30-gauge endodontic irrigating needle (SybronEndo, Orange, CA).

The canals were dried using paper points (Sure-endo, Gyeonggi-do, South Korea), and the fit of the master cone was confirmed. The root canals were then obturated using the lateral compaction technique with a resin-based sealer, ADSeal (Meta Biomed, Chungcheong Buk-do, South Korea), and gutta-percha (Sure-endo, Gyeonggi-do, South Korea). Radiographs in both mesiodistal and buccolingual views were taken with an intraoral sensor (Ez Sensor HD) to verify the quality of the root canal obturation ( Fig. 2 ). Excess gutta-percha was removed, and the canal orifice was sealed. The premolars were then kept at 100% humidity and 37 °C for 7 days to allow the sealer to fully set.

**Fig. 2:**
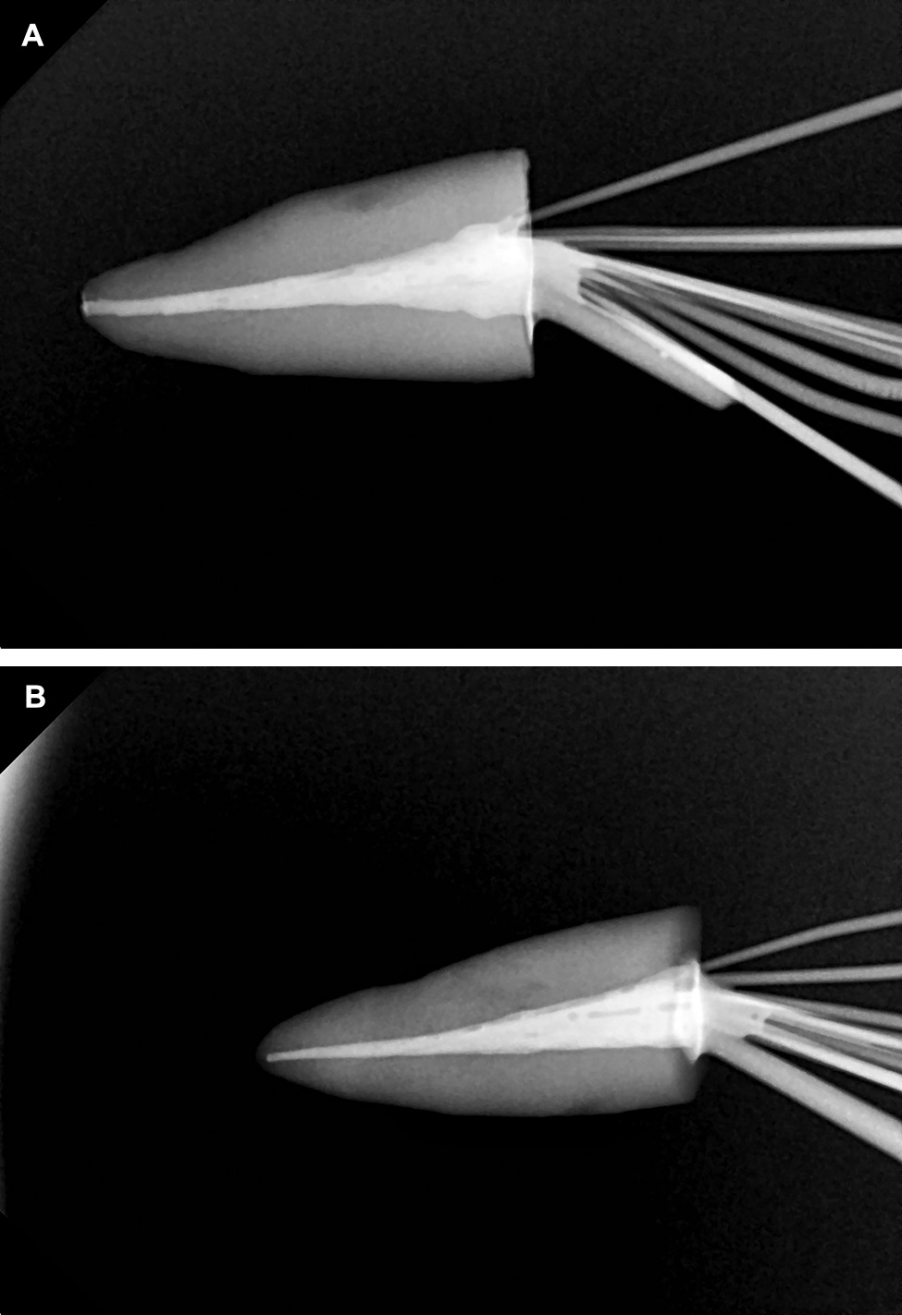
Periapical radiographs that ensure good obturation. ( **A** ) Buccolingual direction, and ( **B** ) mesiodistal direction.

### Retreatment Procedure

After incubation, numbers were assigned to the samples and randomly distributed among the three groups using the Randomization.org website. The sample was divided into three groups, each containing 15 teeth. All retreatment procedures were performed by a single practitioner (T.A.).

#### Group 1: Continuous Rotational Motion Using ProTaper Universal Retreatment

The ProTaper Universal Retreatment system (Dentsply Maillefer, Ballaigues, Switzerland) was utilized, comprising D1 (30/0.09) for the coronal third, D2 (25/0.08) for the middle third, and D3 (20/0.07) for the apical third. Retreatment was performed using the Tri Auto ZX2 device (J Morita, Kyoto, Japan) in continuous rotation at 500 rpm and 1 N·cm torque, following the manufacturer's guidelines. A crown-down approach with pecking motions was applied until the WL was reached. Retreatment was deemed complete when the D3 file reached WL without further retrieval of filling material, or when instrument separation occurred. 10 Each canal was irrigated with 5 mL of distilled water during the procedure. 18

#### Group 2: Reciprocating Motion Using WaveOne Gold

The WaveOne Gold Primary file (25/0.07 variable taper; Dentsply Maillefer, Ballaigues, Switzerland) was employed using the VDW Silver motor (VDW GmbH, Munich, Germany) set to WaveOne All mode. The file was operated with a gentle in-and-out motion, limited to four strokes per cycle, and minimal apical pressure was applied. After each cycle, the file was cleaned to remove accumulated debris. Retreatment was considered complete once the file reached the WL without further retrieval of filling material or upon file separation. 10 Each canal was irrigated with 5 mL of distilled water during the procedure. 18

#### Group 3: Optimum Torque Reverse Motion Using ProTaper Universal Retreatment

The ProTaper Universal Retreatment system (Dentsply Maillefer, Ballaigues, Switzerland) was utilized, comprising D1 (30/0.09) for the coronal third, D2 (25/0.08) for the middle third, and D3 (20/0.07) for the apical third of the canal. Instrumentation was performed using the Tri Auto ZX2 handpiece (J Morita, Kyoto, Japan), configured to OTR mode at 500 rpm and 1 N·cm torque, in accordance with the manufacturer's recommendations. A torque setting of 1 N·cm was specifically selected to enable early activation of the OTR mechanism, thereby facilitating assessment of the system's behavior under low-load conditions. 7 The crown-down technique and peaking motions were employed until the full WL was reached. Retreatment was considered complete when the D3 file reached the WL without the ability to remove additional obturation material or upon instrument separation. 10 Each canal was irrigated with 5 mL of distilled water during the procedure. 18

In all groups, the irrigation needle (EndoTop, China) was placed as deeply as possible into the canal without binding, but no deeper than the predetermined WL.

Each canal was instrumented with a new file corresponding to the specific motion kinematics used. If an instrument fractured during retreatment, the procedure was stopped immediately, and the separation was documented.

### Collection of Apically Extruded Debris

Extruded debris and irrigant (distilled water) were gathered in a vial, where the tooth was secured above it using an individually fabricated rubber stopper, as described by Myers and Montgomery. 19 The apex was suspended within a vial. A second bottle held the debris collection device during canal preparation, preventing direct contact with the collection vial. To equalize the atmospheric pressure inside and outside the bottle, a 25-G needle was used to create an opening in the rubber stopper. The bottle was obscured to prevent the practitioner from seeing the root apex during canal preparation. Upon completion of the preparation, each tooth was detached from the receiving vial. The external root surface was then rinsed with 1 mL of distilled water within the receiving vial to collect debris adhering to the external root surface 20 ( Fig. 3 ). The receiving vials were then incubated at 70 °C for 5 days to allow the distilled water to evaporate before weighing the dry debris. An electronic analytical balance A200S (Sartorius Analytic, Goettingen, Germany) with an accuracy of 0.0001 g was used to weigh the receiving vials containing the debris. Three consecutive weight measurements were obtained for each vial with a variance of less than 0.0002 g, and the mean value was calculated. The dry extruded debris weight was determined by subtracting the empty vial weight from the vial containing the debris. Premolars that experienced instrument separation were not included in the evaluation of extruded debris quantity because of the cessation of preparation, preventing them from reaching the apex.

**Fig. 3:**
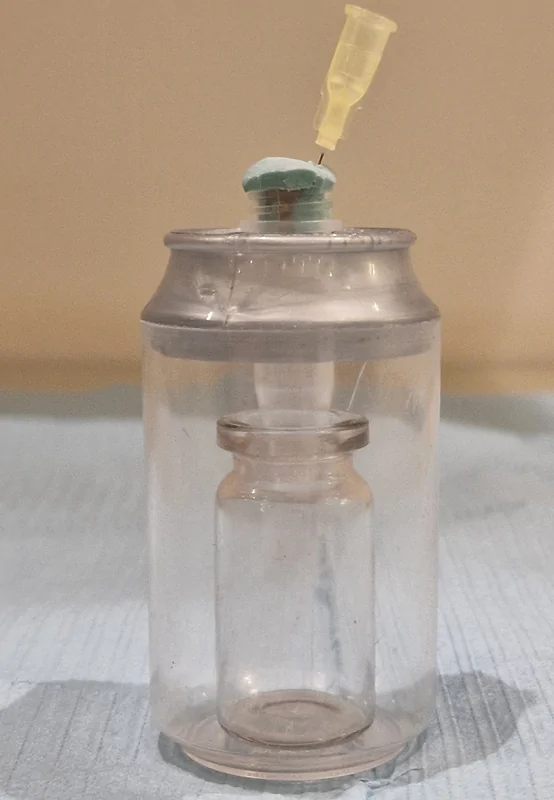
Apically extruded debris collector device.

## Calculation of Residual Obturation Material

After separating the teeth from the rubber stopper, two longitudinal grooves were made on the lingual and buccal surfaces using a 0.26-mm Speedy Ceramic separating disk (ACTEON, United Kingdom). The teeth were then split using a sharp spatula. 15 Each half of the tooth was imaged using a digital camera under a stereomicroscope (Meiji, Japan) at 20× magnification ( Fig. 4 ). The images were processed using ImageJ software (a Java-based image analysis program advanced by the National Institutes of Health and the Laboratory for Optical and Computational Instrumentation, University of Wisconsin) to assess the amount of residual obturation material in three sections: Coronal, middle, and apical thirds 21 ( Fig. 5 ).

**Fig. 4:**
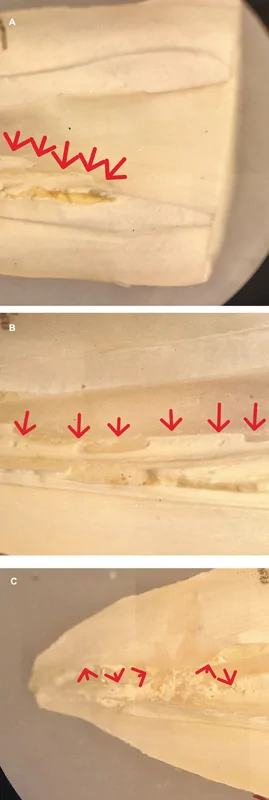
Residual obturation material in the canals (red arrows). **(A)** Coronal third, **(B)** middle third, and **(C)** apical third.

**Fig. 5:**
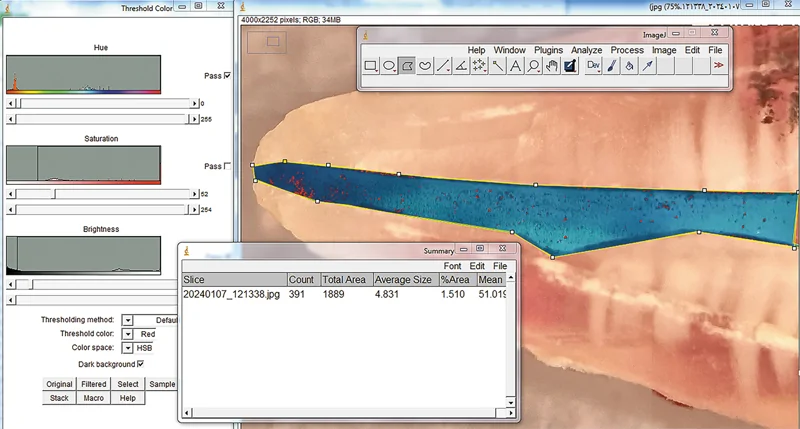
Images analyzed using ImageJ software.

The evaluation was performed by calculating the surface area covered by the obturation material and the total area of each third, then determining the percentage of remaining canal obturation material in each section. Teeth with a separating instrument were excluded from the assessment of the effectiveness of obturation material removal, as the remaining material in these cases was considered to be overestimated.

## Statistical Analysis

The collected data were tabulated and analyzed using the Statistical Package for the Social Sciences (Version 13; IBM SPSS Inc., Chicago, IL). The comparison between groups and intragroups regarding residual obturation material amount in the three levels and the apically extruded debris amount was performed using the one-way ANOVA test. Moreover, the frequency of instrument separation during the retreatment process among the three groups was assessed using the chi-square test. The level of significance was set at α = 0.05.

## Results

The sample consisted of 45 mandibular premolar roots with standardized conditions, evenly distributed into three groups for the retreatment procedures.

### Frequency of Instrument Separation

Regarding instrument separation, six instruments were separated in the OTR group. Table 1 details the frequency of instrument separation and the results of the chi-square test.

**Table 1:** The frequency of instrument separation and the results of the chi-square test

Motion kinematics	Frequency of instruments separation		Chi-square value	*p* -Value a
	Not separated	Separated		
Continuous rotational motion	15	0	13.846	0.001 b
Reciprocating motion	15	0		
OTR motion	9	6		

Abbreviation: OTR, optimum torque reverse.

a Chi-square test.

b Significant differences.

Table 1 also shows that the files in the OTR group exhibited a statistically significant tendency to separate compared with those in the other two groups.

The cases in which instrument separation occurred were not replaced, as doing so would have introduced selection bias and resulted in unequal opportunities across systems. Furthermore, the frequency of instrument separation can be considered an indirect indicator of the limited effectiveness and clinical suitability of systems prone to frequent fracture. This approach is consistent with the methodology adopted by Madarati et al, in which the studied groups were not equal in number due to instrument separation. 15

### The Apically Extruded Debris Amount

Table 2 illustrates the mean, standard deviation, minimum, and maximum values of the extruded debris weight in milligrams, and the one-way ANOVA test result.

**Table 2:** Descriptive analyses of the extruded debris weight in milligrams and the one-way analysis of variance test result

Motion kinematics	Tooth number	Mean	Standard deviation	Minimum	Maximum	*f* - Value	*p* -Value a
Continuous rotational motion	15	0.92	0.35	0.4	1.6	2.309	0.114
Reciprocating motion	15	1.25	0.47	0.4	2.1		
OTR motion	9	1.61	1.43	0.9	5.4		

Abbreviation: OTR, optimum torque reverse.

a One-way analysis of variance test.

There were no statistically significant differences among the three groups in extruded debris weight.

### Residual Obturation Material Amount in the Three Levels

Table 3 presents the mean, standard deviation, standard error, minimum, and maximum values of the residual obturation material ratio for file motion kinematic and canal third, along with the one-way ANOVA test results.

**Table 3:** Descriptive analyses of the residual obturation material percentage and the one-way analysis of variance test result

Third	Motion kinematics	Tooth section number	Mean	Standard deviation	Minimum	Maximum	*f* -Value	*p* -Value a
Coronal third	Continuous rotational motion	30	0.22	0.16	0.006	0.689	1.629	0.203
	Reciprocating motion	30	0.20	0.16	0.006	0.617		
	OTR motion	18	0.30	0.24	0.073	0.953		
Middle third	Continuous rotational motion	30	0.24	0.15	0.03	0.565	1.031	0.361
	Reciprocating motion	30	0.26	0.22	0.006	0.84		
	OTR motion	18	0.32	0.22	0.019	0.708		
Apical third	Continuous rotational motion	30	0.32	0.17	0.01	0.679	1.003	0.372
	Reciprocating motion	30	0.39	0.27	0.015	1		
	OTR motion	18	0.41	0.27	0.113	1		
Overall	Continuous rotational motion	90	0.26	0.16	0.006	0.689	2.599	0.07
	Reciprocating motion	90	0.28	0.23	0.006	1		
	OTR motion	54	0.34	0.24	0.019	1		

Abbreviation: OTR, optimum torque reverse.

a One-way analysis of variance test.

There were no statistically significant differences among the three groups in the amount of residual obturation material.

## Discussion

The main objective of endodontic retreatment is to alleviate the signs and symptoms associated with infectious processes by completely removing the previous root canal obturation materials, re-establishing apical patency, and reducing the microbial load within the canal after implementing an appropriate cleaning and disinfection protocol, which contributes to the healing process of periapical tissues. 20 The previous process should be accompanied by minimal apical extrusion of preparation debris, which can cause postoperative pain, periapical inflammation, and delay healing, 22 and by a reduced risk of instrument separation.

Previous studies examining the impact of continuous versus RM on the efficacy of removing root canal obturation materials and the amount of apically extruded debris have yielded conflicting results. With the emergence of this new motion (OTR), the present study aimed to evaluate the differential impact of this debatable motion on the aforementioned variables, which directly affect the success rates of retreatment and posttreatment pain in the clinical situation. In addition, Pedullà et al 7 found that the OTR motion increases cyclic fatigue resistance. Moreover, a previous study by the authors 18 found that canal retreatment took longer with WaveOne Gold Primary files than with instruments operating in continuous rotation (OTR) or OTR motion. Therefore, the present study aimed to evaluate the effectiveness of OTR motion in removing root canal obturation materials, the amount of apically extruded debris, and the incidence of instrument separation as a complication, compared with CRM and RM.

The immersion of the teeth in 5.25% NaOCl for 1 minute was performed solely to remove organic surface residues and ensure standardized external root surface cleanliness before the experimental procedures. This short exposure was not intended for full dentin deproteination, which requires significantly longer exposure times, as demonstrated by Hu et al. 16 Instead, a 1-minute rinse is widely considered sufficient for surface decontamination without inducing structural alterations in dentin or affecting subsequent measurements. The protocol was, therefore, selected to standardize the samples while avoiding unnecessary chemical changes associated with prolonged NaOCl exposure.

The selected premolars had straight canals (curvature less than 10 degrees) to facilitate standardization of canal anatomy, as mandibular premolars often exhibit a Type I canal configuration according to Vertucci's classification, which simplifies splitting. 23 Crowns were removed to standardize the WL and the approximate amount of root canal obturation material within the sample, and to eliminate the influence of variables such as crown anatomy and access cavity design, leading to more reliable results. 24 The WL was set to be 1 mm short of the apical constriction to reduce the amount of apically extruded debris.

Canals were obturated using the lateral condensation technique with AD Seal sealer, as it is the most commonly used technique. 25

Since one of the objectives of this study was to evaluate the effectiveness of different techniques for removing root canal filling materials, no additional canal preparation was performed to avoid any influence of further preparation on the study outcomes. As the manufacturer's instructions for the ProTaper Retreatment Universal system indicate that the instrument reaching the apical third is the D3 file (#20), the WaveOne Gold Primary file (#25) was selected, as it is the reciprocating instrument closest in size to the D3 file. 24

Instrument separation was monitored throughout retreatment to assess the mechanical performance of each motion kinematic. Given that the OTR motion is still relatively new and under investigation, it was essential to observe the risks of instrument separation associated with this kinematic to provide a comprehensive understanding of the new technique. According to the current findings, no instrument separation occurred during either CRM or RM. However, instrument separation occurred during OTR motion in 6 of 15 cases. These differences were statistically significant, and the null hypothesis was rejected. These results may be attributed to the continuation of the instrument's motion within the root canal obturation mass in an RM with a resultant rotation in the CW direction. The instrument is subjected to additional stresses from root canal obturation materials, which hinder its advancement and cause it to rotate while the coronal part continues to rotate, leading to separation due to torsional fatigue. This result may also be explained by the increased pressure applied to the instruments in the absence of a gutta-percha solvent. It is worth noting that this factor should be taken into consideration in future studies, either by using different solvents or by replicating the current research under initial canal preparation conditions rather than during retreatment. These results are consistent with those reported by Tantiwanichpun and Kulvitit, 10 who indicated separation of ProTaper Next, designed for CRM, during retreatment with adaptive motion.

Notably, the samples that underwent instrument separation in the OTR group were excluded from the other parts of the study to prevent bias in favor of this group. This approach is similar to that used in previous studies. 10 15

Because the amount of apically extruded debris is one of the variables evaluated in this study, the modification suggested by Bürklein and Schäfer 20 was applied to the Myers and Montgomery model, 19 which is widely accepted for measuring apically extruded debris. One limitation of this method is the absence of opposing physical pressure provided by periapical tissues, which could limit the amount of apically extruded debris. Due to the absence of opposing pressure in this design, gravity may have caused the irrigating solution to overflow. This is a known drawback of laboratory designs lacking periapical resistance, as previously noted by Myers and Montgomery. 19

It has been suggested that the resistance of periapical tissues be simulated using floral foam. However, this material might absorb some of the irrigating fluids and debris when used as a barrier. 20 Therefore, no attempt was made in this study to simulate periapical tissue resistance.

According to the current findings, there were no significant differences in the amount of apically extruded debris during retreatment among the three groups, and the null hypothesis was accepted. Despite differences in design, cross-sectional shape, and motion kinematics between the ProTaper Universal Retreatment and WaveOne Gold systems, both systems produced equal apical extrusion of debris when used sequentially with CRM and RM. For the ProTaper Universal Retreatment system, the D1 and D2 files may have removed the majority of the coronal and middle thirds' obturation material, thereby reducing the volume of obturation material and potentially affecting the amount of apically extruded debris. Conversely, the off-centered motion of the WaveOne Gold files might have created space along the canal, facilitating coronal extrusion of obturation material, and reducing its volume, potentially affecting apical debris extrusion. The OTR motion did not affect the amount of apically extruded debris when used with the ProTaper Universal Retreatment system because the cumulative effect of the reciprocating part of the motion was compatible with the design of the file intended for removing obturation material when rotated in a CW direction, thus helping to remove the majority of the coronal obturation material, similar to the continuous rotation's effect on pushing the debris apically. The file design, independent of the motion kinematics, may be the main factor affecting the amount of apically extruded debris.

The current findings agree with Yılmaz and Özyürek, 11 who found no differences in the amount of apically extruded debris during retreatment with CRM, RM, or adaptive motion. However, the results differ from those of Labbaf et al, 26 who reported that RM resulted in greater apical extrusion of debris than continuous rotation during retreatment. This result may be attributed to the centered design of the Reciproc system used in their study, as well as to the difference in the range of RM compared to WaveOne Gold, which potentially acts as a piston for apical extrusion of debris. Moreover, the current findings differ from those of Abd el-ghaffar et al, 12 who reported that continuous rotation motion resulted in greater apical extrusion of debris than OTR during canal treatment. The discrepancy may be attributed to the use of different file systems in the previous study and to the fact that the earlier research focused on primary root canal preparation, whereas the current study investigated retreatment procedures. The findings also differ from those of Al Omari et al, 13 who reported that OTR motion significantly reduced apical debris extrusion compared with continuous rotation and reciprocation. This discrepancy may be attributed to the fact that their study focused on the primary preparation of curved canals, whereas the current study investigated retreatment procedures.

Additionally, the research results differ from those of Bürklein and Schäfer, 20 who found that continuous rotation with the ProTaper Universal system resulted in less apical extrusion of debris than with the Reciproc and WaveOne systems. This result may be due to the larger size of the WaveOne and Reciproc files used in their study compared to the latter file used in continuous rotation.

Furthermore, the research results differ from those of a previous study by Karataş et al, 27 who found that adaptive motion was superior in terms of apically extruded debris compared with CRM and RM. This result may be attributed to the design of the TFA file, which incorporates the file's engagement during the transition between CRM and RM and the angles of RM, potentially reducing apical pressure and thereby reducing apical extrusion of debris.

The amount of residual root canal obturation material was investigated using the vertical splitting method of the teeth. Although this method is destructive to the sample, it provides a direct view and, therefore, a better identification of the residual obturation material in the canal, especially with adequate magnification. Furthermore, it is more effective than two-dimensional radiographic images in assessing the amount of residual obturation material. 28 While some residual obturation material may be lost during splitting, this can be minimized by carefully splitting the samples without detaching the obturation material from the canal walls. 24

For more accurate measurements, digital imaging techniques were used for the halves of the teeth under a stereomicroscope at 20× magnification to gather a clear view of the residual obturation material, as recommended by Bhagavaldas et al. 29 The surface area still covered by the obturation material and the surface area that appeared clean was calculated using ImageJ software, and the percentage of residual obturation material was then computed. This method is effective because it is easy to use and maintains a consistent distance between the device and the measured object, enabling standardized imaging conditions. Additionally, it allows isolation of dentin color, helping to avoid manual error in determining the area covered by residual obturation material and facilitating the identification of tiny particles of residual obturation material. 21

The findings of this study suggested no statistically significant differences among the three groups in the effectiveness of removing root canal obturation materials, and the null hypothesis was accepted.

The similarity in the removal of root canal obturation materials may be attributed to the measurement and tapering of the D3 file, the last instrument used to remove root canal obturation materials in the ProTaper Universal Retreatment system, compared to the Primary file of the WaveOne Gold system. Additionally, the reciprocating portion of the OTR motion did not affect the amount of remaining root canal obturation material when used with the ProTaper Universal Retreatment system, as its cumulative effect with CW rotation was compatible with the file's design for cutting during CW rotation, thereby not affecting its cutting efficacy.

No motion kinematic associated with the instrument could remove all root canal obturation materials due to anatomical variations and the inability of any system to contact all canal walls. 30 31 32 33 The primary factor affecting the effectiveness of removing root canal obturation materials may be the supplementary preparation using a larger retreatment file than the initial root canal preparation size. 24 34 35

The current findings align with those of Rios et al 24 and Faus-Matoses et al, 30 who found that both WaveOne and ProTaper Universal Retreatment files were equally effective. On the other hand, the current results differ from those of Matta et al, 36 who reported that continuous rotation was superior to RM for removing root canal obturation materials. Moreover, it differs from the Koçak et al's 37 study, which showed that RM was superior to CRM for removing root canal obturation materials, compared with continuous rotary motion and hand files combined with Gates–Glidden drills. This may be attributed to differences in file size: They used a large WaveOne file (40) compared to the D3 file from the ProTaper Universal Retreatment system. 20

Furthermore, the current results differ from previous studies comparing the effectiveness of adaptive motion, RM, and CRM in removing root canal obturation materials, which found adaptive motion superior. 2 38 This may be attributed to the larger range of RM angles determined by the Elements Motor preparation device each time, potentially causing instrument binding during rotary motion within the root canal obturation material mass and removing the obturation material upon transitioning to the larger range of RM.

One drawback of this study is the difference in the alloy composition of the instruments used for retreatment. The ProTaper Universal Retreatment system is made of conventional Ni-Ti alloy, whereas the WaveOne Gold system is made of heat-treated NiTi alloy.

Further investigations can be conducted on this topic using advanced evaluation methods for residual obturation materials in the canal, such as CT scans. Additionally, this study could be extended to include curved teeth or teeth with more complex root canal systems, such as molars, to enhance understanding of the mechanisms of action of the three-motion kinematics in retreatment procedures. Furthermore, it is suggested that well-controlled clinical studies be conducted to assess postoperative pain levels and the time required by the three systems to perform endodontic retreatment procedures.

## Conclusion

In light of the limitations of this *in vitro* study, the motion kinematics during retreatment did not affect the effectiveness of gutta-percha removal or the amount of extruded debris. However, the OTR motion resulted in file separation during retreatment. Continuous rotation and reciprocation seem to be safer motion kinematics.
